# Modern precipitation of hydrogenetic ferromanganese minerals during on-site 15-year exposure tests

**DOI:** 10.1038/s41598-020-60200-5

**Published:** 2020-02-26

**Authors:** A. Usui, H. Hino, D. Suzushima, N. Tomioka, Y. Suzuki, M. Sunamura, S. Kato, T. Kashiwabara, S. Kikuchi, G.-I. Uramoto, K. Suzuki, K. Yamaoka

**Affiliations:** 1 Centre for Advanced Marine Core Research (CAMCR), Kochi University, Nankoku, 783-8502 Japan; 2 Japan Oil, Gas and Metals National Corp., Japan (JOGMEC), Tokyo, Japan; 3 Sumiko Resources Exploration & Development (SRED) Co. Ltd., Tokyo, Japan; 4 Japan Agency for Marine-Earth Science and Technology (JAMSTEC), Yokosuka, Japan; 5 School of Science, The University of Tokyo, Tokyo, Japan; 6 Present Address: RIKEN, Tsukuba, Japan; 7 Geological Survey of Japan (GSJ, AIST), Tsukuba, Japan

**Keywords:** Marine chemistry, Physical oceanography, Mineralogy

## Abstract

Redox-sensitive metallic elements, Mn and Fe, are oxidized in deep sea waters and form abundant ferromanganese crusts and nodules on the world’s ocean floors at ultraslow rates of growth. This process of oxidation and the mechanism of precipitation are yet unknown. In this paper, the results of the first successful, long-term, on-site experiment of mineral precipitation that ascertains modern, ongoing hydrogenetic deposition of oxide materials from normal seawaters at water depths of 900–4500 m of geologically active and inactive environments are presented. We succeeded in the *in-situ* precipitation experiment on the sea floor and characterized the precipitates using high-resolution and submicron-scale chemical, mineralogical, and structural analyses. The installed artificial plates of glass, ceramics, and plastic yielded spread-out particles of sizes varying from one to a few micrometers in diameter, of coccoid-like irregular shapes, with a maximum of 1,000–10,000 individual particles/mm^2^/year after 12–15 years of exposure. The results indicated a continuous substantial growth of the hydrogenetic minerals if both Mn and Fe are supplied to the bottom waters. The mineralogical, chemical, and structural properties of the precipitates are similar to those of the natural precipitates on the seabed that are made up of hydrogenetic ferromanganese crusts and nodules, together with settling sediments, suspended hydrothermal particles, or microbial precipitates from cultivated Mn-oxidizing bacteria. Our work presents new realistic insight into proposed genetic models of marine hydrogenetic ferromanganese deposits in modern diverse ocean environments.

## Introduction

Marine ferromanganese oxide deposits have formed the major sink of manganese and iron in global geochemical cycles in the aquatic environment for the last million years. A simple model of the precipitation of ferromanganese minerals forming hydrogenetic crusts and nodules was proposed mainly based on selective chemical leaching^1–5^, assuming dissolved forms of manganese and iron in sea waters of the oxygen minimum zone (OMZ) followed by oxidation and co-precipitation of the metals. The process of oxidation and the mechanism of precipitation in the oceans are yet to be deciphered. We succeeded in using well-controlled deep-sea platforms (manned and unmanned vehicles) for sampling and *in-situ* measurements on and near seafloors, and using high-resolution electron microscopy, energy-dispersive X-ray spectroscopy, and electron diffraction method on the recent precipitates.

Radiochemical, paleontological, and paleomagnetic age models have indicated an average growth rate of hydrogenetic ferromanganese crusts of several to 10 mm/Myr^4–8^. Monitoring of this ultraslow process of precipitation of ferromanganese minerals in modern oceans is considered unrealistic; hence, the only attempt made was a trial on the Hawaii Island sea area^9^. The detailed physicochemical mechanisms of oxidation, precipitation, and growth are poorly understood and we have no knowledge of the chemical or mineralogical form of ferromanganese precipitates in seawater either on the surface or within the crusts or nodules. This experiment is crucial for understanding the origin of metals, the paths of oxidation in global geochemical cycles, and selective absorption of metals by colloidal material.

On-site precipitation experiments and laboratory tests of precipitation and absorption have been performed to understand the processes and mechanism of precipitation of manganese and iron in some terrestrial environments, soils, hot and cold springs, desert varnish, meadows, and seafloor hydrothermal vents^10,11^. However, such experiments have never been performed in deep-sea environments.

Since 2001, institutions including Kochi University (KU), the Japan Agency for Marine-Earth Science and Technology (JAMSTEC), Japan Oil, Gas and Metals National Corporation (JOGMEC), and Geological Survey of Japan (GSJ), have focused on geochemical and mineralogical characterization of hydrogenetic ferromanganese deposits in the northwest Pacific Ocean seamounts using remotely operated vehicles (ROVs) and manned submersibles (Shinkai 6500, JAMSTEC). We succeeded in collecting unbroken precipitation samples and on-site data by carefully deploying and recovering the experimental devices at exact points. Furthermore, we recorded real-time positions, depths, topography, photos, and physicochemical parameters (conductivity, temperature, water depth, and dissolved oxygen) throughout path taken by the ROV.

Geological, mineralogical, chemical, and microstructural analyses were performed on the recovered artificial slides with seabed samples. The samples were observed using optical microscopy, scanning electron microscopy (SEM) equipped with energy-dispersive X-ray spectroscopy (EDS), transmission electron microscopy (TEM), confocal laser microscopy, X-ray and electron diffraction, and X-ray fluorescence spectroscopy.

## Installation of Devices and Recovery

The first on-site precipitation or exposure experiment was designed decades ago by mineralogists at the Massachusetts Institute of Technology; however, it was unsuccessful. For absorption tests, they placed some membrane-sealed synthetic Mn minerals in sea water150 m above siliceous ooze sediments and nodules at a depth of 3000–3500 m^12^. A second exposure test was conducted at a depth of 800–1985 m at the Cross Seamount; the results were recovered 42 months later^9^. Small irregular-shaped amorphous aggregates of Fe and Mn oxide, proved to be the weathered materials of volcanic glass and Mn carbonate rocks were observed but showed no data of mineralogy or morphology. A hydrothermal Fe oxide is described^13^ as a modern precipitate over a recent volcanic glass, which is abundantly available at the Loihi Seamount^14,15^ and East Pacific Rise^16–18^. The divalent Fe oxide minerals in volcanic rocks may result in Fe oxide as a product of oxidation in seawater, which is assumed to be the precursor or initial material for marine ferromanganese deposits. All these experiments were challenging and generated valuable data and insight; however, no evidence of on-going mineral precipitation was found.

**Figure 1:**
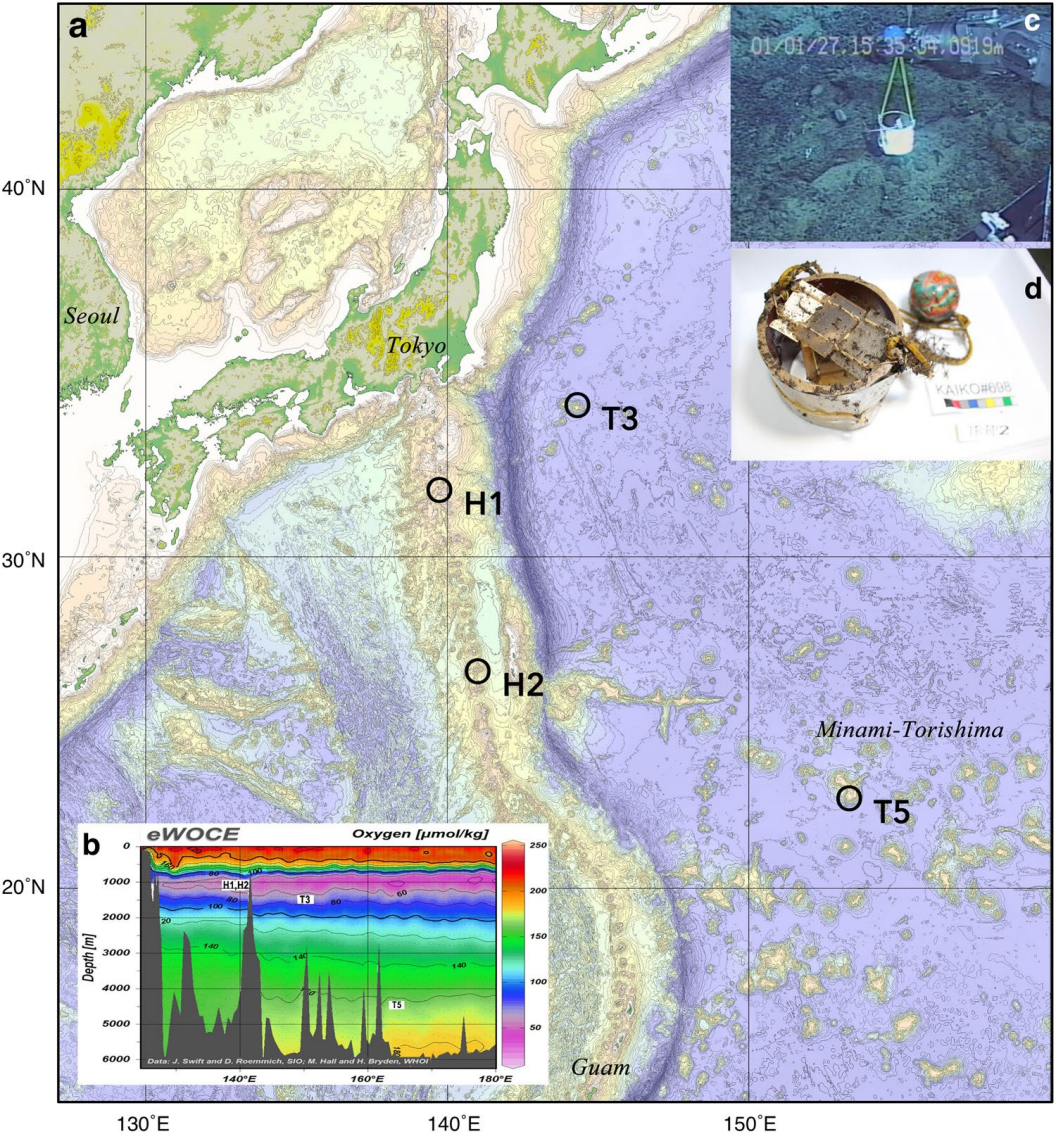
Location and setting of experiments. **(a)** Sites of exposure tests. (**b**) Dissolved oxygen profile and approximate depth of the sites (eWOCE). (**c**) Locations of the experiment devices. (**d**) Example of a recovered device.

Exposure experiments were performed at four locations (two active and two inactive seamounts) near the western margin of the Pacific plate and the eastern margin of the Philippine Sea plate (Fig. 1). Installation and recovery were performed by ROVs, namely Hyper-Dolphin 3 K, Kaiko 7 K, and the manned submersible Shinkai 6 K, (JAMSTEC). The first installation of experimental devices started in 2001 (Fig. 1), and the final recovery was completed in 2017. Five devices (with slides) were deployed on the sea floor, and four were recovered (Table 1) almost exactly at the same location that they were deployed at. Successful deployment to the set position and safe recovery of devices were performed by high-accuracy positioning systems. During the first Shinkai 6 K dives (JAMSTEC Cruise YK01-04), we set up five devices around at 1000 m depth on the slopes of an active submarine volcano, the Kita-Bayonaisse knoll (station H1; 918 m water depth), and the Kaikata Seamount (station H2; 1054 m water depth). Low-temperature hydrothermal water discharge and related massive Mn and Fe oxide deposits were identified at nearby locations^19–21^. The exposure experiments at station H1 lasted for a period of 11 years and 9 months, beginning May 2001 through March 2013, and the experiments at station H2 were completed in March 2016, lasting for 14 years and 7 months.

**Table 1:** Location, water depth, occurrences, and results of on-site experiments.

Station	H2	H1	T5	T3
Area	Kaikata smt.	Daini-Bayonaise knl.	Takuyo-Daigo smt.	Takuyo-Daisan smt.
Geology	Active submarine volcano	Active submarine volcano	Creaceous Guyot	Creaceous Guyot
Base plates	plastic/glass/ceramics & synthetic buserite	plastic/glass/ceramics & synthetic buserite	plastic	glass
Topography	floor of western slope	southern slope of knoll	southern ridge of smt	northern ridge of smt
Cruise set	6 K#606 (YK01-11)2001 Mar	6 K#611 (YK01-11)2001 Mar	KK#684 (KR16-01) 2016 Jan	KK#743 (KR17-07C) 2017 Apr
Cruise recovery	KK#698 (KR16-13) 2016 Oct	HPD#1494 (NT13-05) 2013 Mar	KK#702 (KR16-13) 2016 Sept	KK#786 (KR18-11C) 2018 Aug
Time exposed	15 yrs +7 months	11 yrs + 9 months	7 months	16 months
Bottom material	volcanic sand	volcanic rock outcrop	rock outcrop	rock outcrop
W. Depth (m)	1054	918	4478	1480
Temperature (°C)	4.0	4.4	1.5	2.7
Dissolved Oxygen (mL/L)	1.5	1.2	3.5	0.9
Salinity (‰)	34.3	34.3	34.7	34.4

The second installation was conducted on the slopes of two inactive flat-top seamounts Takuyo-Daigo (station T5, 4478 m water depth; foot of the seamount) and Takuyo-Daisan (station T3, 1480 m water depth; shoulder of the seamount). The flat-top morphology is the result of subsidized carbonate reefs^22^. Station T5 was located at 1500 m deep near the flat top seamount, about 2000 km to the southeast of Tokyo, whereas T3 was only 350 km far from Tokyo. The age of the substrate volcanic rock is 100 Ma^23^, and most of the outcrop is covered with a maximum of 10 cm of hydrogenetic ferromanganese crusts in both the seamounts^4^. Underlying material, age of the substrate, and topography are similar for stations T5 and T3. The CTD measurement showed a strong oxygen minimum zone around 900 m at station T5 and 1400 m at T3. The average bottom current rate is fairly high, about 0.5 m/s, indicating that the seamounts are in well-oxygenated conditions. The exposure time was set around 1 year since our previous experiment showed significant evidence of deposition of ferromanganese minerals over 12–15 years at stations H1 and H2.

The metal flux or mass accumulation onto nearby ferromanganese crusts was calculated as 0.08–0.10 g Mn/cm^2^/Myr, 0.05–0.10 g Fe/cm^2^/Myr^24^ for the last million years, which are about two magnitude orders smaller than that for the normal pelagic sedimentation in deep-sea basins^25^. Be-10 isotope data indicated an approximate average growth rate of the hydrogenetic crusts to be 4–7 mm/Myr^5^.

At each station, one or two slides (28 × 38 × 2 mm^3^) were rinsed using 6N hydrochloric acid after cleaning with boiled water and ethanol, fixed by thin polypropylene rope, and suspended 10 cm above the seafloor inside a heavy ceramic tube of 10 cm diameter. Three types of material, i.e., polyethylene, glass, and ceramics were used for plates. The devices were recovered carefully using a manipulated version of the submersible or ROV, usually kept in a water-tight aluminum box, and retrieved on deck. Immediately after recovery, microbial cells on the slides were fixed by 3% paraformaldehyde in 1 x PBS (pH 8.0) for 12 h at 4 °C and stored in 100% ethanol at −80 °C until fluorescent microscopic analysis.

The recovered plates were air-dried at room temperature and covered with evaporated carbon, osmium, or gold for observation using the SEM to determine any modern precipitates on the exposed plate surfaces. Furthermore, the entire surface of the plate was observed using high-resolution field-emission scanning microscope (JEOL JSM-7001FA) and analyzed using EDS. Part of the precipitates was analyzed in detail using TEM (JEOL JEM-ARM200F) with EDS.

A 100 mL bottle filled with a suspension of synthetic buserite (Na_2_O·6MnO_2_) crystals^12^ was deployed at each site for cation exchange and metal absorption experiments. The results of this chemical analysis after exposure during this experiment are reported elsewhere.

### Morphology and chemistry of precipitates

**Figure 2:**
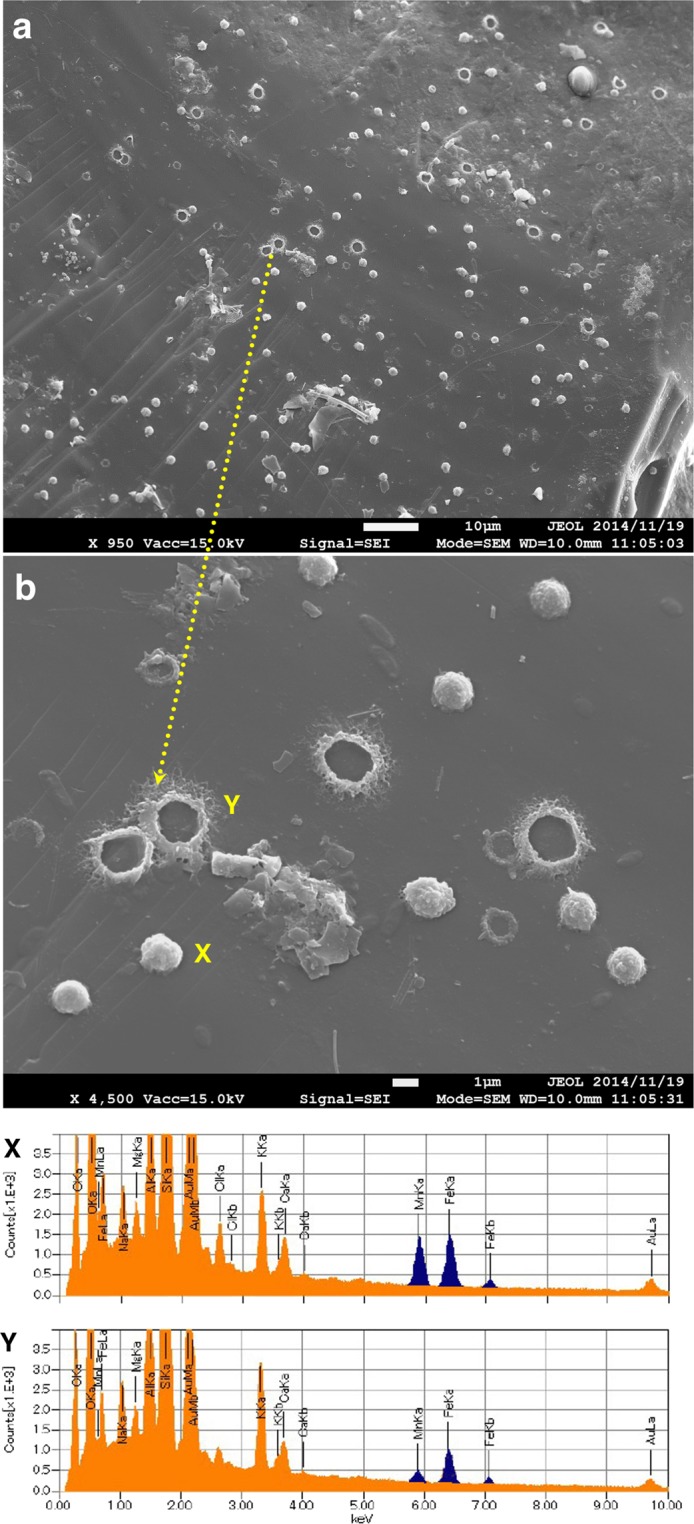
SEM micrographs and EDS chemistry of Fe-Mn precipitates. (**a**) Spread-out particulate ferromanganese aggregates on the slides (station H1). (**b**) Chemical analysis (EDS) of the two particulate precipitates of different morphology (X: coccoid and Y: donut).

We investigated the surfaces of all plates using SEM and found abundant modern precipitates of ferromanganese oxides of various morphology on all types of plates; the devices were deployed in various geological environments, including modern active submarine volcanoes in the subduction zone (stations H1 and H2) and inactive flat-top seamounts of the Cretaceous age later covered with reef limestone and pelagic carbonate sediments (stations T5 and T3). Additionally, the precipitates were observed across a range of water depths (918–4478 m) and redox potential (DO; 0.9–3.5 mL of oxygen [O_2_] per liter). The size of the particulate matter was 1–2 µm or more; the shape is mainly coccoid-like, discoidal, or rarely irregular. Occasionally, the sphere was broken into a donut shape at stations H1 and H2 (Fig. 2a,b). We observed several independent particulate aggregates directly on the surface of plates from both stations, together with planktonic siliceous and calcareous skeletons, benthic agglutinated foraminifers, volcanic rock fragments, clays, and organic tissues. The major morphology comprised coccoid-like spheres with a diameter of 1–2 µm; however, the ferromanganese minerals in some cases appeared hollow. Another morphology was elongated irregular aggregates that were often connected. Particulate morphological type was common at the two stations.

**Figure 3:**
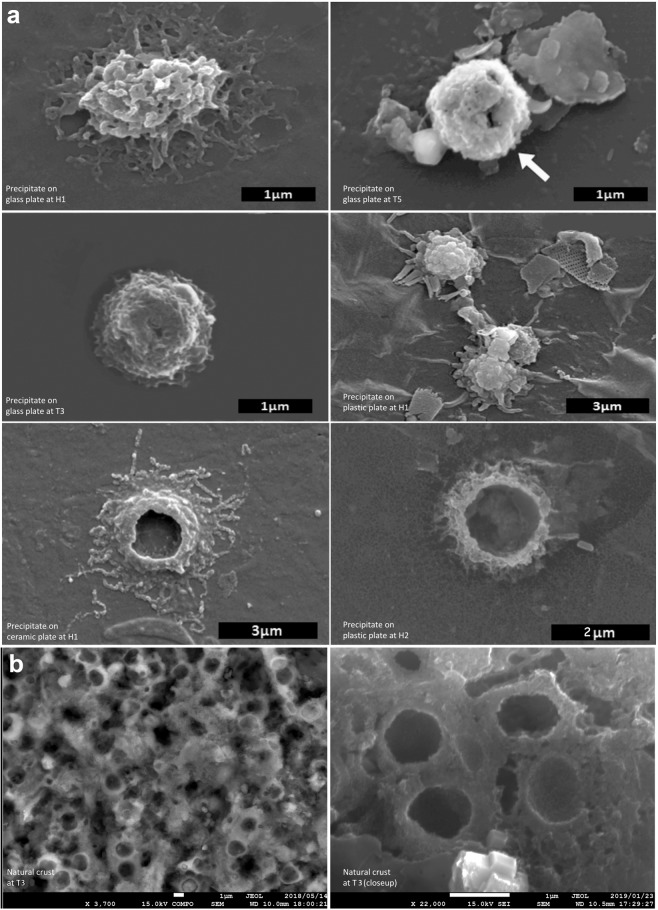
SEM micrographs of Fe-Mn precipitates. (**a**) Variable morphology of particulate Fe-Mn oxide under SEM from ferromanganese crusts from different seamounts and water depths. (**b**) Similar morphology and size of particulate aggregation within hydrogenetic ferromanganese crusts at station T3.

In the offshore inactive old seamounts Takuyo-Daigo (station T5) and Takuyo-Daisan (station T3), we observed ferromanganese oxide particulate aggregates with irregular morphology on the exposed glass slides (exposure of 1 year or less), similar to those observed from stations H1 and H2, although they were relatively scarce (Fig. 3a). Furthermore, this characteristic of the accumulation of ferromanganese oxide particles is common in typical natural hydrogenetic ferromanganese crusts (Fig. 3b).

The chemical property of the ferromanganese oxide was determined using field-emission SEM combined with EDS (JOEL JSM-7001FA) after normal ZAF correction, which provides ratios of elements (e.g., Fe/Mn) rather than absolute contents. The semiquantitative EDS analysis indicated that the Fe/Mn is 0.8 ± 0.2. The SEM-EDS analysis showed that the Fe/Mn ratio of the particulate ferromanganese oxide aggregate of station H1 was 0.6–1.7 (avg. 1.01; n = 126) and that of station H2 was about 0.7–1.1 (avg., 0.89; n = 164). The Fe/Mn ratio of the ferromanganese oxide aggregates on the plate of station T5 (4478 m water depth) was 0.71–1.27 (avg.; 0.98; n = 13) at the Takuyo-Daigo seamount and was 0.20–0.40 (avg. 3.3; n = 5) at T3 of Takuyo-Daisan seamount.

**Figure 4:**
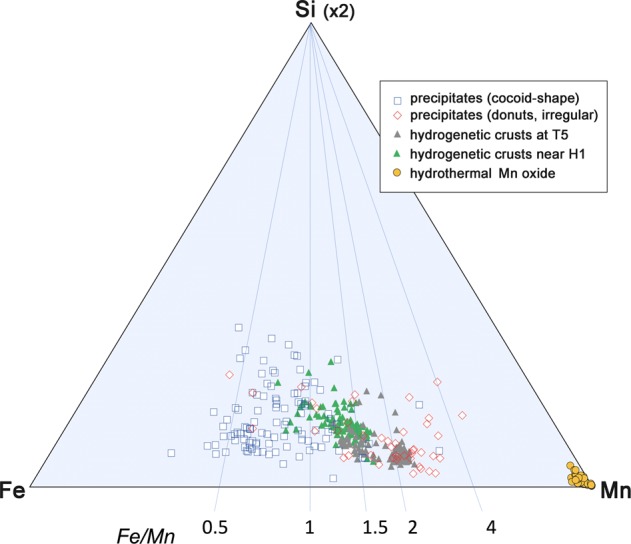
SEM-EDS chemical analysis of particulate oxides. Note the limited range of Fe/Mn ratio between 0.5 and 3 (open squares) overlapping the range of natural hydrogenetic ferromanganese crusts (solid triangles). The data plots for the precipitates from H1 and for the hydrogenetic crusts from T5 in comparison.

The Fe/Mn ratio of precipitates on the slides ranged from 0.3 to 1.1 (avg. 0.8), which was wide but showed no significant trends with the type of plates or seamounts. The range and average were close to that of typical hydrogenetic ferromanganese crusts shown in bulk chemical compositions, ranging from 0.64 to 1.27 (avg.; 0.68: n = 1488)^7,26^. The similarity of morphology and chemical composition of natural modern precipitates to that of typical hydrogenetic ferromanganese crusts (Fig. 4) suggests a common origin of those materials. The chemistry of precipitates from the modern submarine volcano is close to natural hydrogenetic vernadite, a poor-crystalline Fe-Mn mineral, and not to the typical hydrothermal Mn or Fe deposit, which is highly fractionated to very high or low Fe/Mn. As shown, the ratio of Fe/Mn of the variable precipitates did not change with locality (i.e., active and inactive), water depth, or material of the plate. The similar chemistry in the ratio of Fe/Mn around unity or smaller suggests that the ferromanganese mineral of the modern precipitate is generally a hydrogenetic vernadite although the metals were supplied originally from hydrothermal water or normal seawater at depths between 1 and 6 km. The rate of precipitation of hydrogenetic ferromanganese deposits was roughly estimated as 0.2–2 mm/Myr from the average number of spherical particles per area in the plates, assuming close packing, and a specific density of 2 for the particles. This rate is comparable to the average growth rate of several millimeters per million years for hydrogenetic crusts.

These results provide evidence of the mineralogical form of initial precipitates in normal deep-sea environments that form hydrogenetic ferromanganese oxide deposits (crusts and nodules). Regardless of the geological setting, water depth, and variable redox potential, the initial precipitate in the normal seawater is Fe-essential vernadite.

### Mineralogy and microbiology

**Figure 5:**
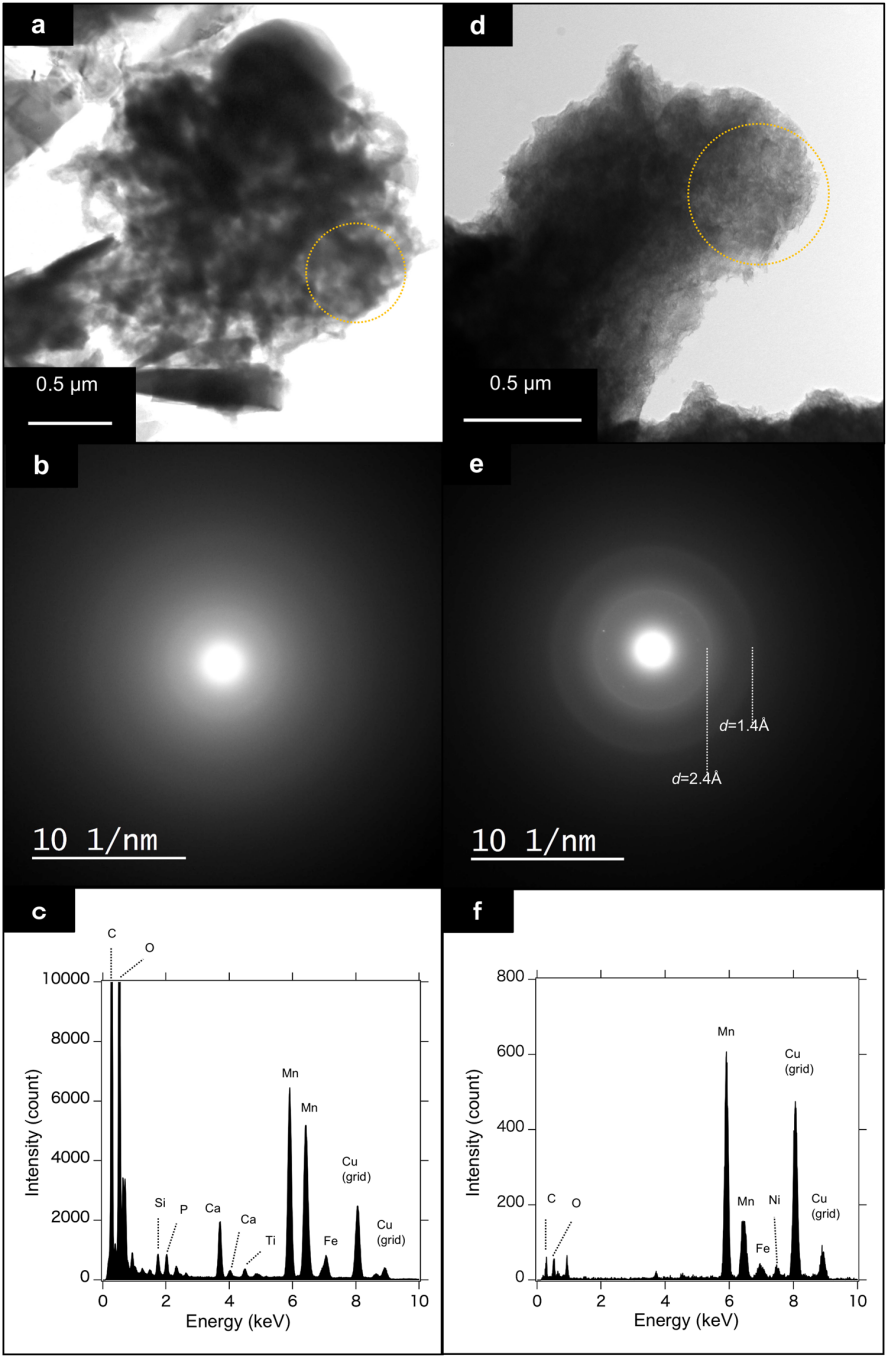
TEM micrograph and electron diffraction patterns of precipitate and natural ferromanganese crusts. Both showing a typical diffuse pattern for the nano-scale poorly crystalline nature of a typical vernadite. (**a**) TEM micgographs, (**b**), SAED pattern, (**c**), ED pattern for the modern precipitates, and (**d**–**f**) for the natural hydrogenetic crusts.

TEM-EDS were applied to characterize the mineralogy of the Fe-Mn compound in the coccoid-shape modern particulates, in comparison with a natural hydrogenetic Fe Mn crust deposit. Among the four sites of variable materials of plates and water depths the chemical property of the major element was similar under SEM-EDS, showing approximately 1.0 Fe/Mn ratio and a 1 to 2 µm-scale spherical morphology. In selected-area electron diffraction (SAED), the precipitate samples did not show any prominent diffraction rings under a very broad diffraction profile, whereas a natural hydrogenetic crust showed faint diffused diffraction rings corresponding to 2.4 and 1.4 Å in *d*-spacings, the diagnostic dual diffused ring pattern of the ferromanganese mineral vernadite (Fig. 5).

Vernadite is a typical Fe-Mn mineral present in hydrogenetic crust and exhibits very low crystallinity and submicron size; consequently, it produces the broad X-ray diffraction lines. The two diffraction lines result from edge-sharing MnO_6_ octahedral sheets intergrown with the isostructural ferrihydrite sheets shows no distinct basal reflection because of highly disordered stacking^27,28^. Such a low crystallinity and intergrown structure is unique to ocean hydrogenetic Fe-Mn oxide deposits, as well as other Fe-bearing Mn dioxides^29^, although mineralogical nomenclature is still controversial.

When modern precipitates were compared with the mineralogy of hydrothermal and early-diagenetic ocean Mn deposits, characteristics of distinct diffraction at the basal 10 or 7 Å *d*-spacings, higher crystallinity, and extremely low Fe content (called as buserite, birnessite or todorokite), and no equivalent diffraction rings were observed in the SAED patterns. The absence of basal diffraction as well as small particle size, low crystallinity, and high Fe/Mn ratios indicate that our modern precipitates are hydrogenetic vernadite. A similar vernadite-like SAED pattern was reported in the micron-size Fe-Mn particles in deep-sea clay sediments^30^, which may have a common nature in depositional processes and environments.

**Figure 6:**
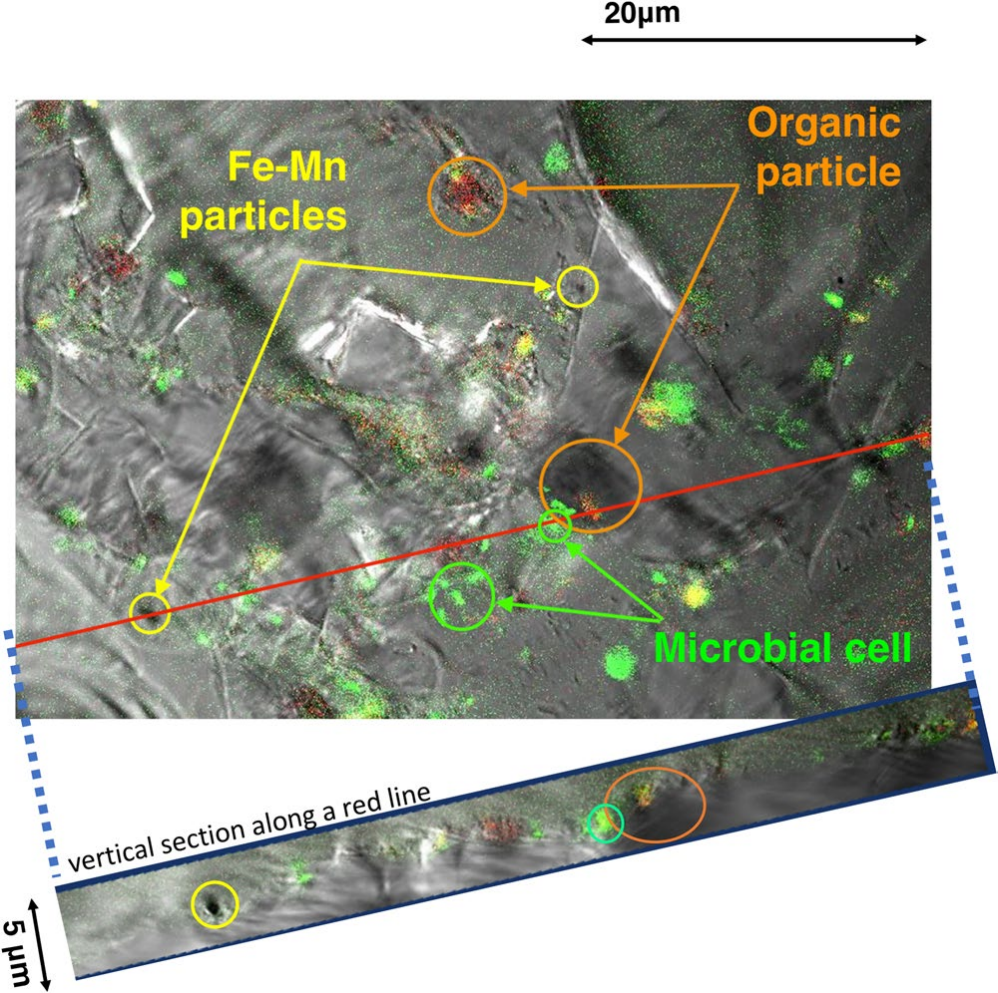
Confocal laser microscope image of the precipitate and microbial cells. The image was obtained after fluorescent staining of a sample and synthesized from a transmitted light image, a blue excitation fluorescence image, and a red excitation fluorescence image. Upper image is horizontal image on the slide and lower image is vertical image along with the red line. The microbial cells (green particles, with green arrows) are associated with organic-like particle (red arrows) but are not associate with the ferromanganese oxide particles (yellow arrows), indicating no evidence of *in-situ* Fe and Mn oxide precipitation with living microorganisms on the slides.

As shown in earlier microbiological studies, some marine and terrestrial microbes can accelerate the oxidation of divalent Mn in soils, hot springs, wells, rivers, meadows, and ocean hydrothermal vents^1,2,31–35^. Microbial effects on oxidation and reduction of Mn and Fe have been proposed in aquatic sediments and aqueous environments. Some kinds of bacteria accelerate the oxidation of Mn^2+^ and produce micrometer-scale aggregates with tissue- or coccoid-like morphology^36,37^. We examined a possible contribution of microbes that promote the precipitation of ferromanganese minerals on the seabed. Confocal laser scanning with fluorescence microscopy showed that distribution of SYBR Green I-stained microbial cells was associated with organic-like particles but not with the modern ferromanganese precipitates on the plates (Fig. 6). This indicates that living microbes may not always play an important role in the oxidation of dissolved Mn on the plates, unlike the marine Mn-oxidizing *Bacillus* sp. strain SG-1^2,38,39^, which positively oxidizes soluble divalent Mn.

Accordingly, we considered a minimal microbial source for the modern ferromanganese oxide precipitate on the glass slide recovered after a 15-year exposure at station H1. Previous reports of DNA analysis of the seawater, rock outcrop, and associated sediments have suggested that most microbes use ammonia–ammonium as energy sources rather than divalent Mn potentiality in the seamount bacterial locality^40–42^. We assume that major microbes do not have positive evidence of accelerated oxidation in this experiments at different water depths.

### Model of formation

Considering the global geochemical cycles of Mn and Fe in oceans, the origin and paths of transportation, mechanism of oxidation, and deposition must be understood. The environments and processes of formation of ferromanganese crusts and nodules have not yet been determined in detail. The earlier Koschinsky and Halbachs’ model^3^ for the formation of hydrogenetic ferromanganese crusts assumes that Mn is dissolved as a divalent ion in the environment of OMZ^21,43^ (reservoir of Mn), and is apt to be oxidized in the lower margin of OMZ, thus forming suspended colloids of Mn dioxide that can easily combine with trivalent Fe oxide.

In contrast, an alternative genetic model was proposed by Usui *et al*.^4^ after detailed on-site observations and measurements of the northwestern seamounts, i.e. Takuyo-Daigo seamount, showing that the ferromanganese oxide was precipitating even within modern OMZ, and hydrogenetic deposits grow at all depths^4,24^. In this model, the modern intermediate layer (OMZ) is not a major reservoir of dissolved Mn but still a place of continuous oxidation and deposition of Mn. The model suggests reconsideration on chemical forms of Mn and Fe in normal sea waters with varying redox potential at the depths.

Thus the first on-site successful exposure experiments on the ocean floor for Mn and Fe oxide precipitation were completed after a 15 year exposure at depths between 900 and 4500 m in active and inactive seamounts. The installed artificial plates yielded spread-out particles of one to a few micrometers in diameter comprising coccoid-like irregular shapes with a maximum 1,000–10,000 individual particles per square millimeter per year. The chemical and mineralogical nature of the modern precipitates proved to be similar to natural hydrogenetic ferromanganese minerals.

The results of analyses on the exposed plates ascertained the proposed model of modern continuous precipitation when both Mn and Fe are supplied to ambient underlying waters. The particulate is thus the smallest unique constituent of ferromanganese crust and nodule deposit of the Neogene to modern age at full water depths of oxic deep-sea environments at all oceans. Hydrogenetic particulate, Fe-Mn oxide forms the piled ferromanganese crusts and nodules at a wide-range of depths including OMZ. Thus the major origins of supply of Mn to marine manganese deposits may be such particulate Fe-Mn oxide but not dissolved Mn. Microbial activity may have served as a potential platform for mineral deposition, but the effect of acceleration may be minimal and should be examined in future.
